# eIF3 controls cell size independently of S6K1-activity

**DOI:** 10.18632/oncotarget.4458

**Published:** 2015-06-29

**Authors:** Katharina Schipany, Margit Rosner, Loredana Ionce, Markus Hengstschläger, Boris Kovacic

**Affiliations:** ^1^ Institute of Medical Genetics, Center of Pathobiochemistry and Genetics, Medical University of Vienna, A-1090 Vienna, Austria

**Keywords:** cell size, eIF3, mTOR, S6K, cancer

## Abstract

All multicellular organisms require a life-long regulation of the number and the size of cells, which build up their organs. mTOR acts as a signaling nodule for the regulation of protein synthesis and growth. To activate the translational cascade, mTOR phosphorylates S6 kinase (S6K1), which is liberated from the eIF3-complex and mobilized for activation of its downstream targets. How S6K1 regulates cell size remains unclear. Here, we challenged cell size control through S6K1 by specifically depleting its binding partner eIF3 in normal and transformed cell lines. We show that loss of eIF3 leads to a massive reduction of cell size and cell number accompanied with an unexpected increase in S6K1-activity. The hyperactive S6K1-signaling was rapamycin-sensitive, suggesting an upstream mTOR-regulation. A selective S6K1 inhibitor (PF-4708671) was unable to interfere with the reduced size, despite efficiently inhibiting S6K1-activity. Restoration of eIF3 expression recovered size defects, without affecting the p-S6 levels. We further show that two, yet uncharacterized, cancer-associated mutations in the eIF3-complex, have the capacity to recover from reduced size phenotype, suggesting a possible role for eIF3 in regulating cancer cell size. Collectively, our results uncover a role for eIF3-complex in maintenance of normal and neoplastic cell size - independent of S6K1-signaling.

## INTRODUCTION

Regulation and maintenance of cell number and cell size represent the most fundamental homeostatic mechanisms to preserve the consistency and the continuity of life in all living beings. Unlike unicellular organisms, in which these processes are controlled by the availability of nutrients, multicellular organisms require additional levels of control depending on distinct organs and tissues. While the regulation of cell proliferation has been extensively studied for decades, the regulation of cell size has so far received much less attention.

Cell growth and proliferation are separable processes [1, 2] – hence, cells may grow without dividing (for example, postmitotic neurons) and may proliferate without growing (for example, the divisions in a fertilized egg). Interestingly, differences in animal size or organ size seem to be genetically determined and primarily reflect differences in cell number, rather than differences in cell size [3]. There is a general agreement that non-dividing adult cells that maintain a constant size are not biosynthetically inactive or lacking growth signals, but in a balanced state of protein synthesis and degradation - resulting in no net change in mass and volume.

Cancer development is a consequence of the loss of the cell's ability to regulate its normal homeostatic activities like growth and survival. Recent discoveries have implicated many key players of growth regulatory mechanisms in tumorigenesis [4]. Interestingly, several regulators of protein synthesis have also been found aberrantly expressed or activated in different types of human cancer [5, 6] and thus targeting of the translational machinery may represent a novel anti-cancer treatment strategy [7].

The best-known example of a key regulatory pathway controlling cell growth is the IGF/AKT/mTOR pathway. Aberrant activation of this pathway may cause additional growth in most cell types tested [8–10]. Most prominently, mTOR integrates a plethora of signaling inputs from external cues that affect cell growth (stress, amino acid level, energy supply and oxygen consumption) and thus acts as signaling nodule for growth and translational control [8]. Accordingly, rapamycin, a potent inhibitor of mTOR-signaling exerts a strong negative effect on cell size. To regulate protein synthesis, mTOR modulates the activity of two important translational regulators, the ribosomal S6 protein kinase (S6K) and the eukaryotic initiation factor 4E (eIF4E) [2, 11, 12]. S6K is responsible for activation of the ribosomal protein S6, and thus for the activation of the ribosome, while in parallel, mTOR controls the binding of the ribosome to mRNA through the cap-binding protein eIF4E by regulating its inhibitor, the 4E-binding protein (4E-BP1) [13, 14].

The role for S6K in cell size regulation has been extensively investigated [15], yet some of the findings have yielded contradictory results. Disruption of S6K1 in drosophila caused a reduction of cell size [16], however mice lacking S6K1 or S6K1 and S6K2 were viable, showed body mass reduction (15–20%), but displayed an intact protein synthesis rate and normal phosphorylation of S6 at S235/236 [17, 18]. S6K1-deficiency reduced cell size of pancreatic β-cells [19] and myoblasts [20] and thus it remains unclear whether other cell types may be affected. Interestingly, siRNA-mediated knockdown of S6K1 in human U2OS cells reduced cell size by only 5% [21] and overexpression of S6K1 increased cell size but rapamycin resistant S6K1 mutants could only partially revert the size reduction induced by rapamycin treatment [2].

These findings suggest a more complex regulation of cell size by mTOR-S6K1-signaling than previously proposed. Recent data suggest a mechanistic model for the dynamic sequence in which the translation preinitiation complex (PIC) is assembled during translational initiation. In this model, a sequential order of events takes place involving signals transduced via the mTOR-S6K1-S6 signaling axis using the eukaryotic initiation factor 3 (eIF3) subunit as a scaffold [22]. eIF3 is a large complex comprising of 13 different subunits, termed a to m, which together represent one component of the 40S ribosomal subunit and of the much larger 43S translation PIC. The functional core of human eIF3 is composed of six subunits (eIF3a, eIF3b, eIF3c, eIF3e, eIF3f, and eIF3 h) of which only eIF3a, eIF3b, and eIF3c are conserved in all eukaryotes and represent core units to which most of the other subunits bind [23, 24]. The subunits are conserved in sequence across species, suggesting a high degree of functional conservation as well. Upon mitogenic stimulus, eIF3b and eIF3c subunits interact with inactive S6K1, which - in turn - is released to activate its downstream targets after phosphorylation by mTOR. Significantly, eIF3 subunits have been frequently found overexpressed in a variety of tumors and cancer cell lines [5, 25, 26]. Ectopic expression of five eIF3 subunits (a, b, c, h and i) has caused *in vitro* transformation of murine NIH3T3 cells [5]. Overexpression of eIF3 subunits a, b, c, h, i and m has been seen in many cancers [27]. Moreover, eIF3b was recently implicated as a prognostic marker of human bladder and prostate cancer [28].

Here, we aimed to investigate the role of S6K1 as a mediator of mTOR-induced cell size control. Given the suggested role for eIF3 as a binding factor of inactive S6K1 and a docking station for mTOR on which it phosphorylates S6K1 [22], we developed an eIF3-deficient cell system to observe the effects of uncoupled mTOR-S6K1 signaling on cell size. Using siRNA-induced depletion of core subunits of eIF3 (eIF3b and eIF3c) in normal and transformed cell lines, we show that loss of eIF3 complex leads to a profound reduction in cell size, despite an increase in S6K1-S6 signaling, in all cell lines tested. Interestingly, the hyperactive S6K1-S6 was rapamycin-sensitive, indicating an upstream regulation by mTOR. PF-4708671, a selective S6K1 inhibitor, readily inhibited the S6K1-response, but was unable to revert the reduced size effect. Finally, we proved that restoration of eIF3 expression efficiently recovered the initial cell size, without affecting p-S6 levels. These results uncover a novel role for eIF3 complex in maintenance of cell size, independently of S6K1-signaling.

## RESULTS

**Figure 1:**
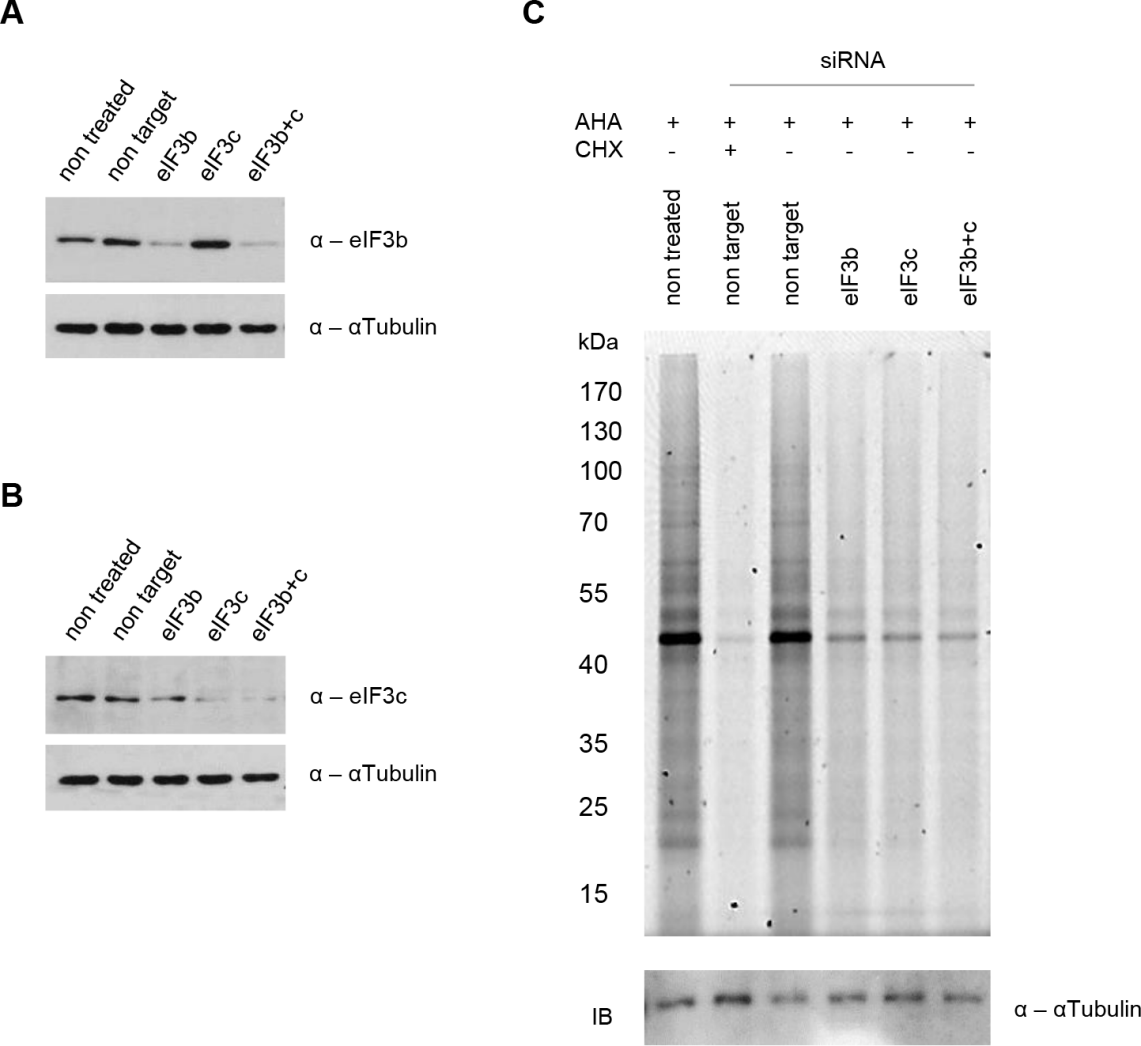
siRNA mediated knockdown of eIF3b and/or eIF3c blocks nascent protein synthesis in IMR-90 cells IMR-90 cells were transfected with specific siRNAs or left untreated as indicated. **A** and **B.** Knockdown efficiency was confirmed by immunoblotting using antibodies specific for (A) eIF3b and (B) eIF3c. αTubulin serves as a loading control. **C.** L-azidohomoalanine (AHA) incorporation was measured after 3.5 hours. Cycloheximide (CHX) was used as a control for total protein synthesis inhibition at a final concentration of 50 μM. Nascent protein synthesis was evaluated by fluorescent scanning of AHA bound Tetramethylrhodamine (TAMRA) at Ex550/Em570. Loading was verified by immunoblotting using αTubulin antibody as a control.

To study the role of eIF3 complex in cell size control, we performed knockdowns of eIF3b and eIF3c in primary, non-transformed, non-immortalized human lung fibroblast cells that carry a normal diploid karyotype (IMR-90 cells). In case that eIF3-complex represents a platform for activation of S6K1 by mTOR, we expected to see a reduction of S6K1-signaling. However, in case that eIF3-complex is essential for retention of S6K1, we expected to observe an increase in S6K1-activity. Depletion of either eIF3b, eIF3c, or both proteins by siRNA, significantly reduced their protein expression after 72 hours (Figure 1A and Figure 1B). We next evaluated whether depletion of eIF3b and eIF3c had any effect on protein synthesis. At 72 hours after siRNA transfection, the global protein synthesis rate was measured during a period of 3.5 hours. *De novo* protein synthesis was strongly diminished upon eIF3b and/or eIF3c knockdown, although this effect was slightly less pronounced compared to control cells treated with cycloheximide for the same period of time (Figure 1C), indicating that translation is strongly dependent on the availability of eIF3b and eIF3c.

**Figure 2:**
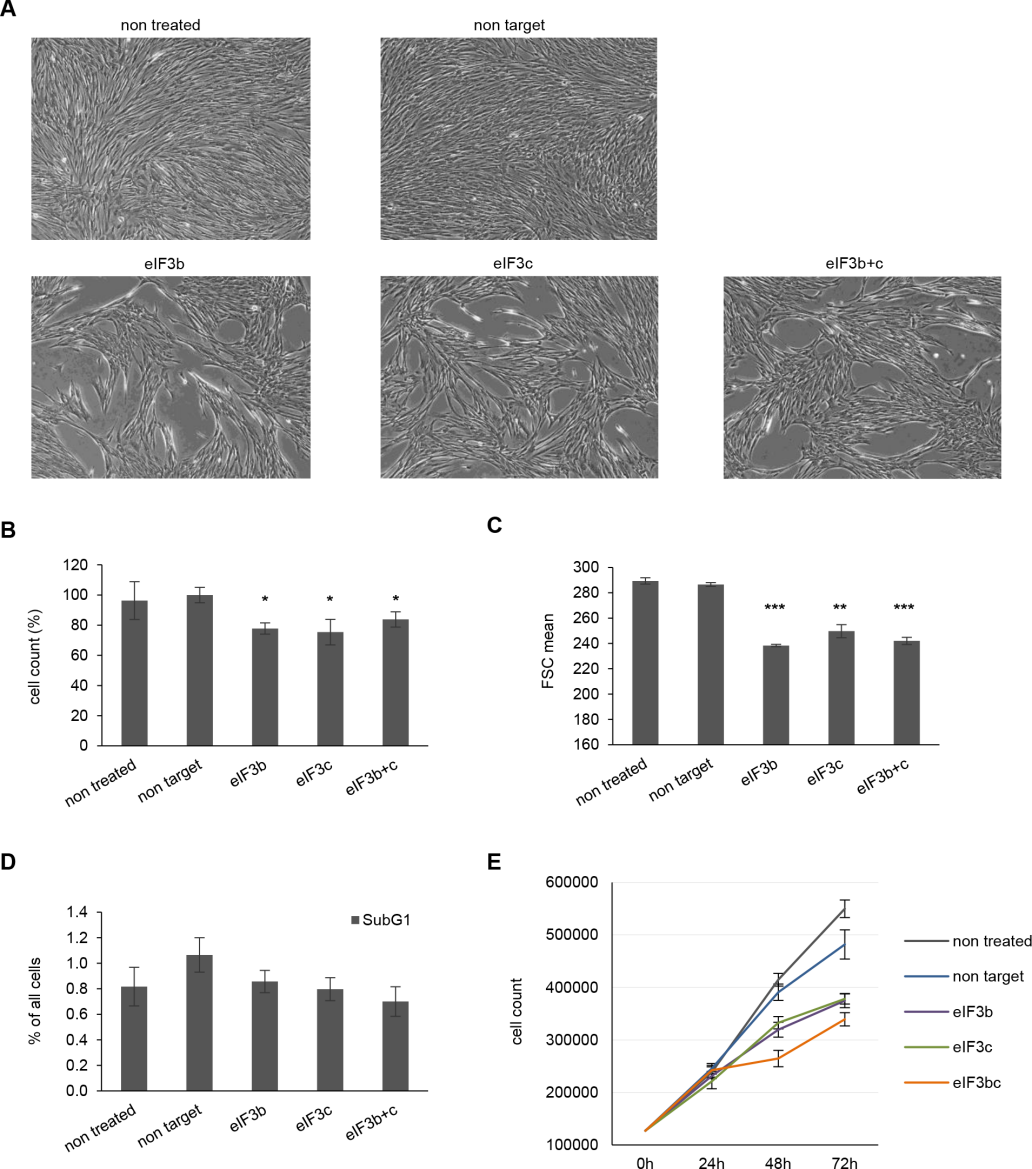
Depletion of eIF3b and/or eIF3c decreases proliferation and cell size of IMR-90 cells **A.** Representative pictures of siRNA treated IMR-90 cells 72 hours post transfection are shown (magnification 4x). **B.** Cell numbers were measured using Casy cell counter at 72 h after transfection. Total numbers were normalized to non-target siRNA-transfected cells. **C.** Cell size was assessed by flow cytometry using the parameter forward scatter (FSC). **D.** Percentage of apoptotic cells (subG1-fraction) was determined by flow cytometry of propidium iodide (PI)-labeled cells at 72 h post transfection. One representative experiment out of two is shown. **E.** Proliferation curves of control and eIF3b-, eIF3c- and eIF3b and c-depleted IMR-90 cells. Cells were transfected and counted at 0, 24, 48 and 72 hours post transfection. (B and C) Figures show means of two independent experiments performed in triplicates. Error bars represent means ± SD.

To determine the biological effects manifested by the loss of eIF3b and eIF3c, we measured cellular density, proliferation, apoptosis and cell size over a period of 72 hours. Interestingly, depletion of either eIF3b or eIF3c equally reduced the cellular density of IMR-90 cells 72 hours after knockdown (Figure 2A). However, a combined knockdown of both proteins could not further reduce this effect. To distinguish whether a decrease in cellular density is a consequence of reduced cell number or reduced cell size, we quantified the total cell number and size using Casy cell counter and flow cytometry. Indeed, eIF3b and/or eIF3c depletion significantly diminished the cell number (−40% on average) and decreased the cell size (−15% on average) 72 hours after transfection (Figure 2B and Figure 2C). Importantly, this difference was not due to a delay in cell cycle (G2/M cells are bigger than G1 cells), since cell cycle measurement revealed no differences in distribution of cell cycle phases between control and eIF3b/c-depleted cells (Figure S1A). However, eIF3b and/or eIF3c-depleted cells showed a significant reduction of size in G0/G1- and S-phases (Figure S1B). To further determine whether a reduced cell number is caused by a lethal phenotype in the fraction of IMR-90 cells that had received eIF3b or eIF3c siRNA, we evaluated the effect of protein knockdown on apoptosis. The amount of apoptotic cells was not altered through the depletion of eIF3b- or eIF3c (Figure 2D). To analyze the effects of protein knockdowns on long-term cell proliferation, we performed time-course measurements of total cell number during a period of 3 days. As illustrated in Figure 2E, single and combined depletion of eIF3b and eIF3c from IMR-90 led to a reduced proliferation that manifested at 48 h and 72 h after transfection with siRNA. To confirm these findings using distinct and independent non-transformed cell culture systems, we reassessed the effect of loss of eIF3b and eIF3c using mouse embryonic fibroblasts (MEFs). Similarly, MEFs devoid of eIF3b and eIF3c protein expression exhibited a dramatically impaired proliferation rate after 72 hours (Figure S1C). Taken together, these data suggest that eIF3b and eIF3c are associated with the cellular control of size and long-term proliferation, rather than affecting the cells' viability.

**Figure 3:**
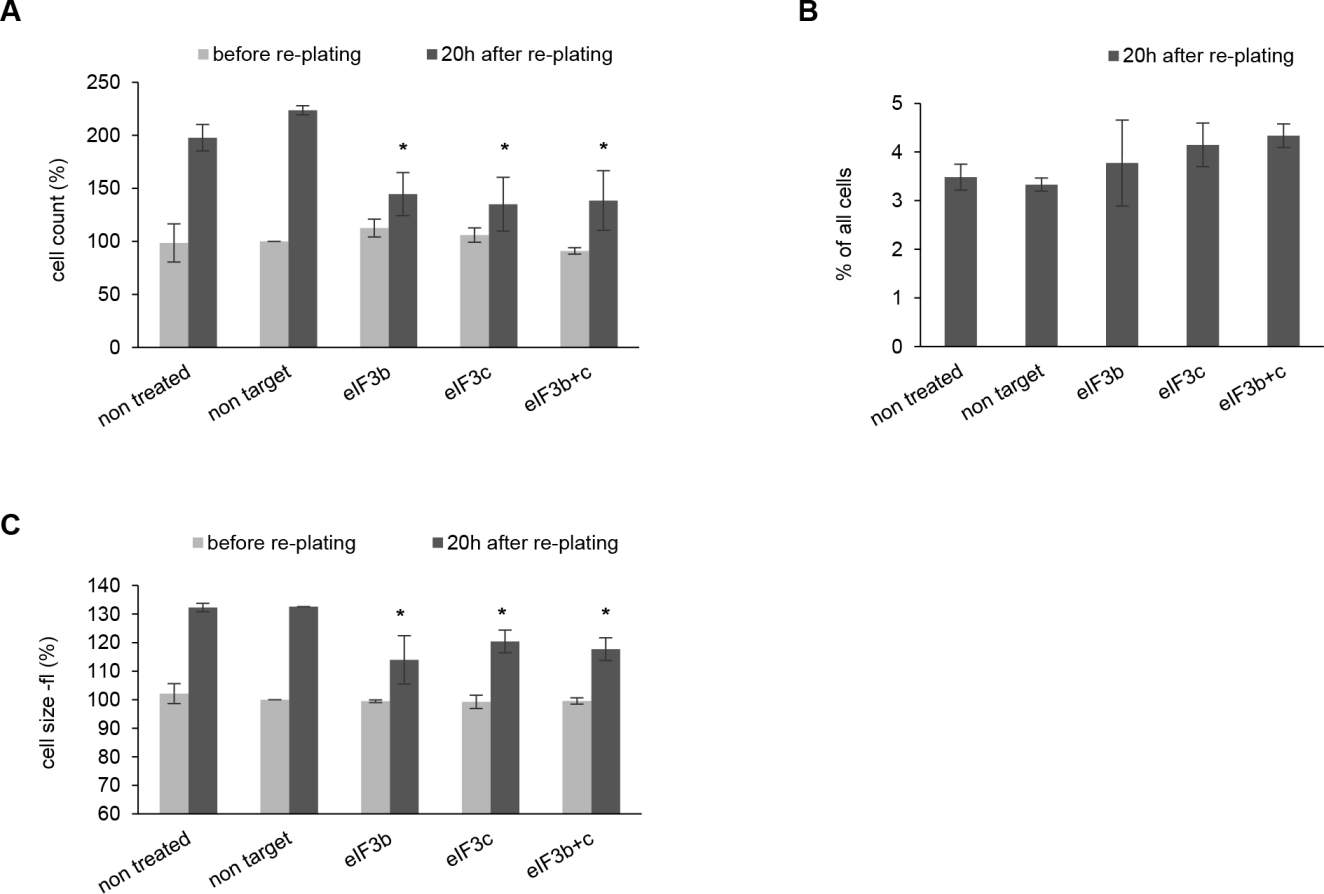
Depletion of eIF3b and/or eIF3c in HEK293 cells causes reduced proliferation and smaller cell size after re-plating of cells **A.** Cells numbers were measured before (72 h after transfection) and 20 h after re-plating. Cell numbers were normalized to non-target siRNA-transfected cells before re-plating. **B.** Percentage of apoptotic cells was evaluated by PI-staining. One representative experiment out of two independent experiments performed in triplicates is shown. **C.** Cell size was determined with Casy cell counter by analyzing cell volume (fl). Cell volumes were normalized to non-target siRNA-transfected cells before re-plating. (A, C) Figures show means of two independent experiments performed in triplicates. Error bars represent means ± SD.

HEK293 cells have been generated by the transformation of human embryonic kidney cells with Adenovirus 5 followed by a prolonged immortalization in culture and now represent a transformed and immortalized cell line with an abnormal (hypotriploid) karyotype [29]. To gain insight into the role of eIF3 complex in a transformed cellular background we depleted eIF3b and eIF3c in HEK293 cells. Interestingly, we did not observe any significant differences in cell number and cell size after siRNA transfection at 72 hours (Figure 3A and Figure 3C, gray bars). We reasoned that this might be due to an increased proliferation rate and a far more dense phenotype compared to IMR-90 cells (data not shown) - which might have unmasked the initial effect. Thus, we harvested the siRNA-transfected HEK293 cells at 72 hours and re-plated them for further 20 hours in order to reactivate the cell cycle. Indeed, HEK293 cells depleted for eIF3b and/or eIF3c showed a strongly reduced proliferation capacity 20 hours after re-plating (Figure 3A, black bars). Also the cell size was obviously reduced after depletion of eIF3b and eIF3c (Figure 3C, black bars). However, knockdown of both core units of the eIF3 complex did not significantly alter the relative amount of apoptotic cells (Figure 3B).

**Figure 4:**
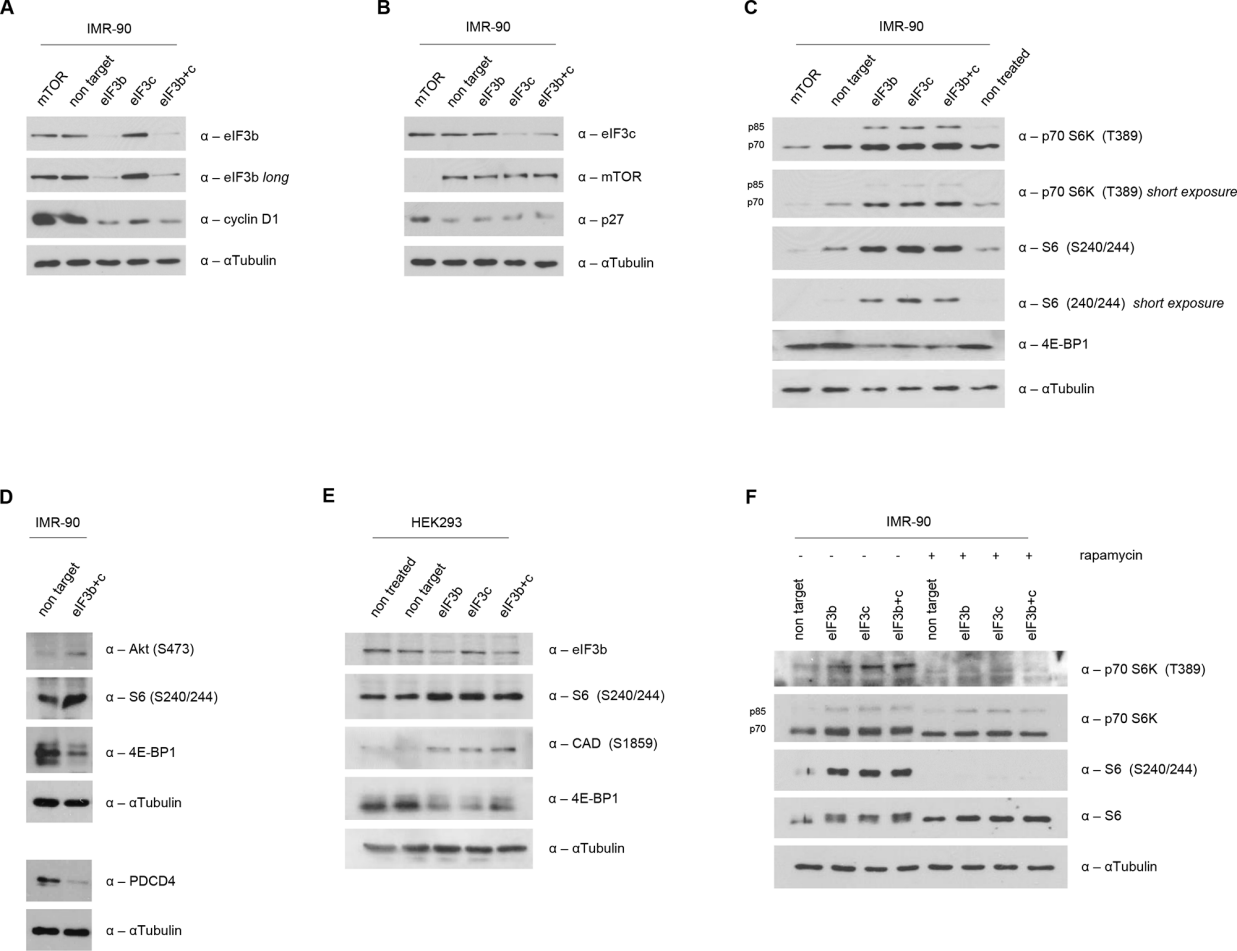
Increased S6K1-activity in eIF3b and/or eIF3c-depleted cells **A.** Western blot analysis of eIF3b and Cyclin D1 was performed in eIF3b and/or eIF3c-depleted IMR-90 cells at 16h after re-plating. **B.** At the same time-point, eIF3c, p27 and mTOR expression levels were determined in eIF3b and/or eIF3c-depleted IMR-90 cells. To avoid any interference in the detection of eIF3b and eIF3c due to similar protein size on the same membrane, same lysates were detected on separate membranes. **C.** Expression levels of mTORC1-specific targets were evaluated by western blotting in eIF3b/c- or mTOR-depleted IMR-90 cells. **D.** mTORC2-specific target p-AKT (S473) as well as PDCD4 expression levels were detected in cell lysates of IMR-90 as indicated. **E.** mTORC1 and mTORC2-specific targets were determined in HEK293 cells by immunoblotting. **F.** 48 hours post transfection, IMR-90 cells were treated with 100nM rapamycin or DMSO for another 24 hours. Total and phosphorylated S6K1 and S6 protein levels were evaluated by immunoblotting. αTubulin was used as appropriate loading control in all panels.

Depletion of eIF3b has previously been shown to strongly decrease the levels of S-phase and G2/M-phase cyclins (cyclin A and cyclin E) in a bladder cancer cell line [28]. To analyze the molecular effects of eIF3b and eIF3c depletion during early stages of cell cycle, we analyzed the relative abundance of the G1-S transition regulators Cyclin D1 and p27Cip/Kip after cell cycle re-stimulation induced by re-plating of IMR-90 cells. Interestingly, in a karyotypically normal primary cell type like IMR-90 cells, depletion of either eIF3b or eIF3c alone, or combined depletion, strongly reduced the amount of cyclin D1 (Figure 4A). In contrast to a recent report using bladder cancer and prostate cancer cell lines [28], we did not observe any increase in p27 cell cycle inhibitor levels (Figure 4B). Depletion of mTOR has recently been reported to inhibit cell cycle progression [30–32]. Interestingly, mTOR knockdown in IMR-90 cells increased the levels of total p27 (Figure 4B), but did not alter the expression of cyclin D1 (Figure 4A), indicating a distinct regulatory effect of the eIF3-complex and mTOR on cell cycle progression.

The mTOR-eIF3-S6K1 signaling nodule has been suggested to have a crucial role in translational regulation [6, 8, 13, 33, 34]. Moreover, the eIF3 complex has been postulated as a scaffold to orchestrate mTOR signaling in a growth factor/serum dependent manner [22]. Briefly, mTOR binds to eIF3, phosphorylates S6K1 thus releasing it from eIF3 in order to subsequently activate its downstream targets. Therefore, we sought to determine the status of the mTOR-eIF3-S6K1 signaling axis in the context of eIF3b and eIF3c depletion. Strikingly, we found that the phosphorylation of p70 S6K1 at T389 was strongly elevated upon eIF3b and eIF3c knockdown conditions in IMR-90 cells (Figure 4C). Importantly, this increase in phosphorylation of p70 S6K1 at T389 was accompanied with a concurrent increase in phosphorylation of its major downstream target, the ribosomal protein S6. Additionally, we found that the total amount of 4E-BP1, another direct target of mTOR and inhibitor of cap-dependent translational initiation, was downregulated upon eIF3b and eIF3c depletion. In contrast and as anticipated, mTOR depletion yielded a downregulation of the S6K1-S6 axis, while the total amount of 4E-BP1 remained unchanged (Figure 4C). To strengthen this finding, we analyzed another direct target of mTOR-activity, p-AKT (S473), which is positively regulated by mTORC2. Indeed, p-AKT (S473) was increased in eIF3b/c depleted cells (Figure 4D, upper panel). Moreover, PDCD4, a negative regulator downstream of S6K1, was strongly decreased (Figure 4D, lower panel) – thus further demonstrating the hyperactivity of S6K1. Time course analysis has revealed an induction of phosphorylated S6 after 48 hours and a reduction of 4E-BP1 levels becoming visible 72 hours after the transfection of cells with siRNA (Figure S2A). Strikingly, the hyperactive S6K1-S6 axis could also be recapitulated in all other human and murine primary cell systems tested here: In HEK293 cells carrying a knockdown for eIF3b and/or eIF3c, we observed an increase in p-S6 and p-CAD (both targets of S6K1) and a decrease in 4E-BP1 levels (Figure 4E). Furthermore, an induction of phosphorylated S6 was also verified in MEFs upon knockdown of eIF3b and eIF3c (Figure S2B). To investigate whether or not mTOR activity is responsible for the hyperactive S6K1-S6 axis, we treated control and siRNA-transfected IMR-90 cells with 100 nM rapamycin for 24 hours. As shown in Figure 4F, rapamycin treatment entirely inhibited both, the phosphorylation of S6K1 at T389 and the phosphorylation of S6 at S240/244, in eIF3b, eIF3c and eIF3b/c knockdown cells. These findings suggest that mTOR may be able phosphorylate S6K1 even in absence of eIF3.

**Figure 5:**
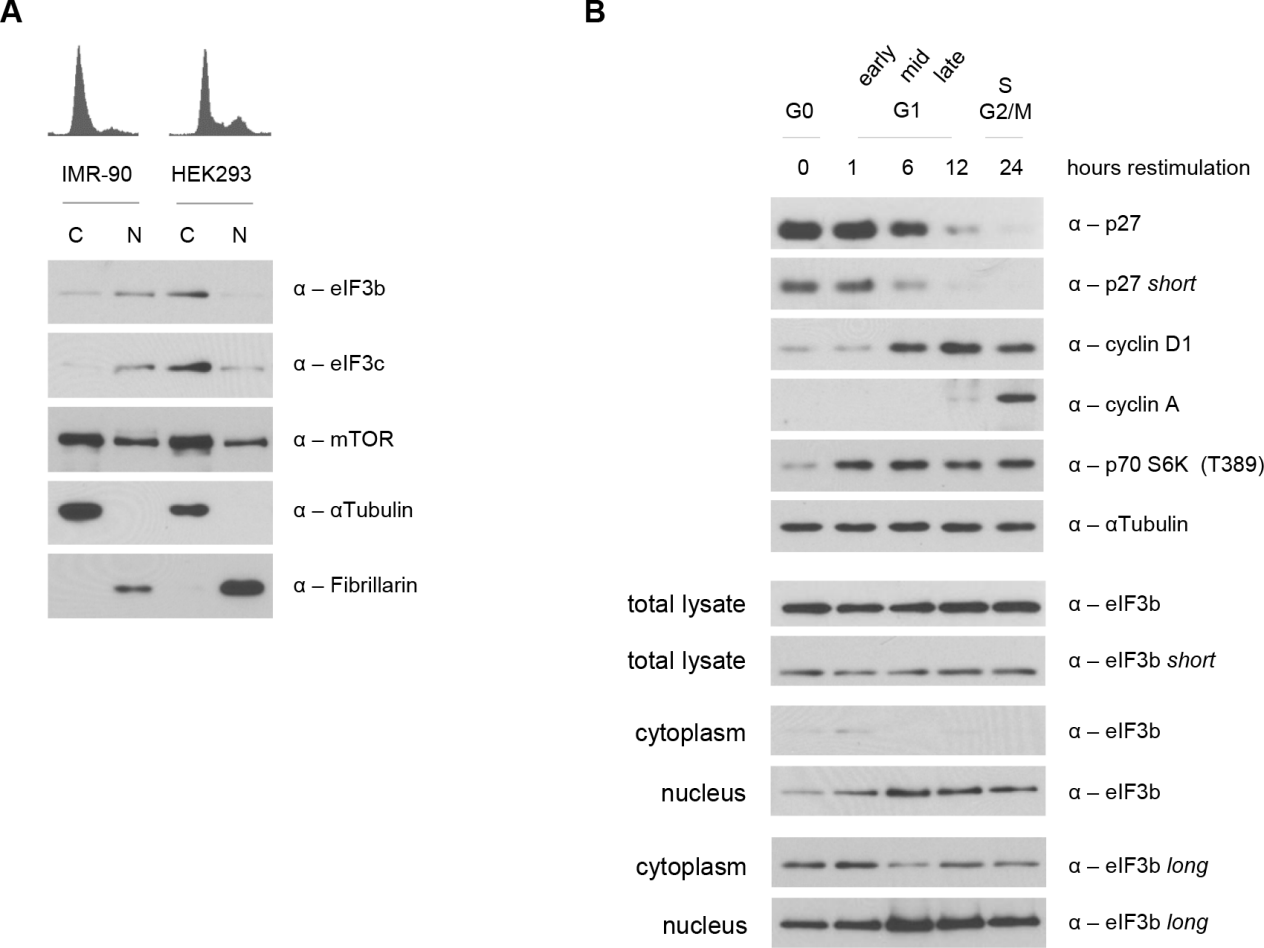
Subcellular and cell cycle dependent localization of eIF3b and eIF3c **A.** Cellular localization of endogenous eIF3b and eIF3c was verified in cytoplasmic (C) and nuclear lysates (N) of IMR-90 and HEK293 cells. Purity of protein fractions was confirmed by the use of antibodies specific for cytoplasmic (αTubulin) and nuclear (Fibrillarin) proteins. **B.** IMR-90 cells were cell cycle synchronized in G0/G1 via serum deprivation and then re-stimulated. Cell cycle regulated proteins like p27, cyclinD1 and cyclinA were used as a control to show the stages of cell cycle progression upon serum re-stimulation (upper panel). Total and subcellular fractions of the same pool of cells were analyzed for eIF3b protein expression at 0, 1, 6, 12 and 24 hours post serum re-stimulation (lower panel).

Recently, we have been able to uncover a cell cycle-dependent nuclear localization of p70 S6K1, mediated by its phosphorylation at T389 via mTOR [33]. To analyze the relationship between hyperactive S6K1-S6 axis in the eIF3-deficient background and the nuclear localization of p-S6K1 T389, we asked whether the absence of eIF3 leads to an enhanced or reduced nuclear trafficking of p-S6K1 T389. Strikingly, we observed an increased presence of S6K1 in the nucleus (Figure S3A). However, this increase in nuclear S6K1 was independent of growth factor signaling since starvation with 0% serum rather increased, than reduced the amount of nuclear p70 S6K1. In contrast, and in line with our previous findings, mTOR-depletion caused a decline in phosphorylated S6K1 levels and prevented its nuclear translocation compared to controls. This suggested that the mislocalization of phosphorylated S6K1 may be a consequence of the loss of its binding factor eIF3 in the cytoplasm. Therefore, we determined the subcellular localization of eIF3b and eIF3c by fractionating nuclear and cytoplasmic extracts of non-transfected logarithmically-growing IMR-90 and HEK293 cell lines. Unexpectedly, we found eIF3b and eIF3c proteins in nuclear and cytoplasmic extracts of both, IMR-90 and HEK293 cells. However, the expression pattern of both proteins in nuclear and cytoplasmic compartments varied largely among the cell lines (Figure 5A). eIF3b and eIF3c remained predominantly nuclear in IMR-90, while being cytoplasmic in HEK293 cells. In contrast, there was no obvious difference in the localization pattern of mTOR between IMR-90 and HEK293 cells. In depth analysis of cell cycle synchronized IMR-90 cells (Figure S3B) revealed that eIF3b localization is tightly regulated during cell cycle progression. We observed increased levels of eIF3b protein during G0 and G1 stages in the cytoplasm. However, between 6 and 24 hours after cell cycle induction (mid G1 to G2/M phase), eIF3b was predominantly localized to the nucleus (Figure 5B). Taken together, these findings suggested that under normal conditions, eIF3 complex may shuttle between cytoplasm and nucleus in a cell cycle dependent manner but that the localization of eIF3 is cell type specific and thus may not fully explain the presence of hyperactive nuclear S6K1 T389 in eIF3-depleted cells.

**Figure 6:**
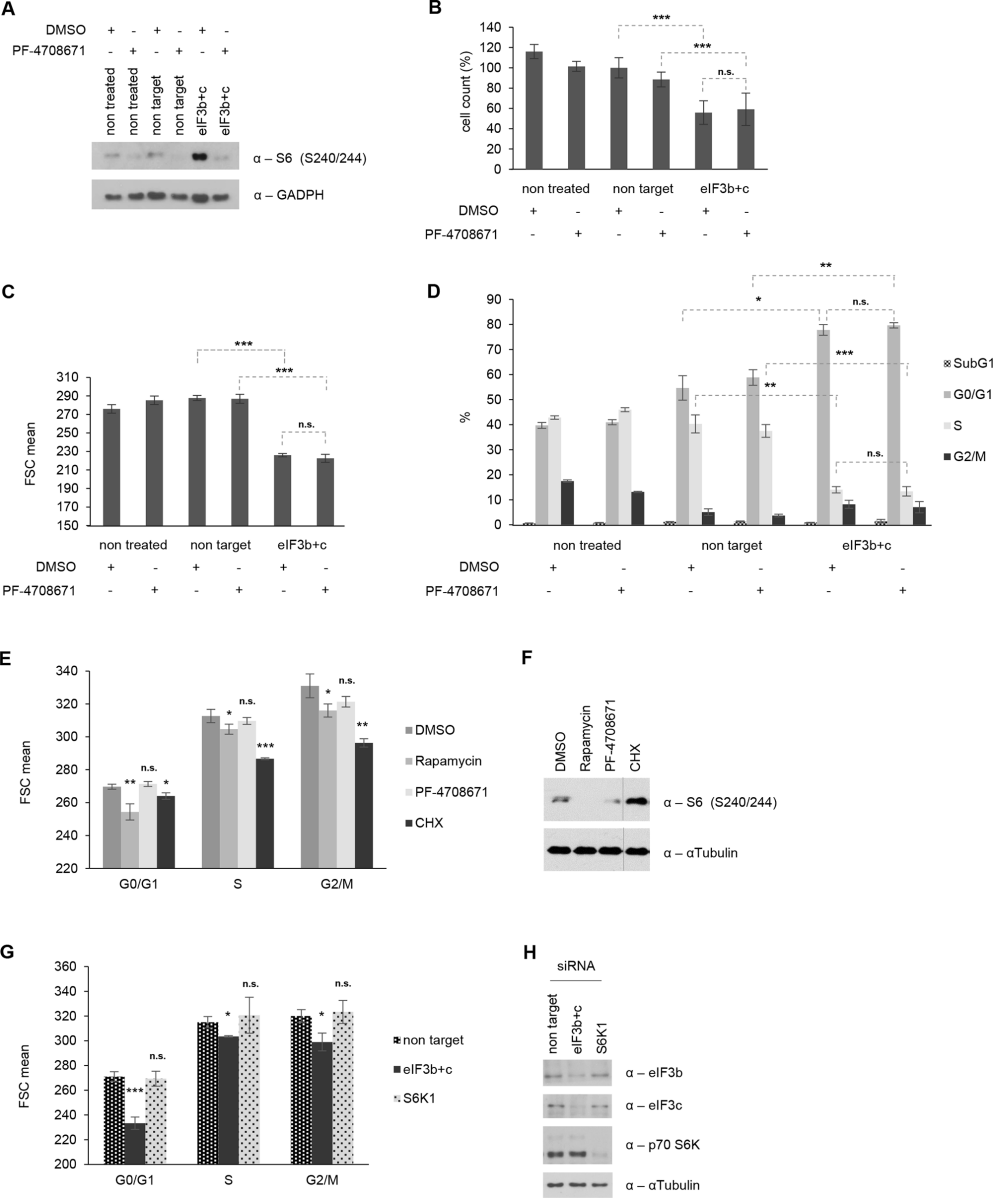
Cell size, proliferation and cell cycle distribution are independent of S6K1 activity **A.** siRNA-transfected cells were re-plated for additional 16 h in presence or absence of S6K1 inhibitor PF-4708671. p-S6 (Ser240/244) levels as a readout are shown. GAPDH was used as a loading control. **B.** Cell number was determined using Casy cell counter. Total numbers were normalized to non-target siRNA-transfected cells. **C.** Cell size was determined by FSC values using flow cytometry. (B-C) Figures show means of three independent experiments performed in triplicates. **D.** Cell cycle distribution including subG1 levels for apoptotic cells. One representative experiment out of three independent experiments performed in triplicates is shown. **E.** IMR-90 cells were treated with DMSO, 100 nM Rapamycin, 10 μM PF-4708671 or 20 μg/ml Cycloheximide for 24 hours. Cell size was measured for each phase of the cell cycle by flow cytometry. **F.** Corresponding cell lysates were immunoblotted for p-S6 (S240/244). **G.** IMR-90 cells were transfected with specific siRNAs for 72 hours as indicated. Cell size was assessed by flow cytometry for each phase of the cell cycle. **H.** Corresponding cell lysates were immunoblotted to confirm knockdown efficiency. (E, G) One representative experiment out of two independent experiments performed in triplicates is shown. Error bars correspond to means ± SD.

eIF3 has been suggested to exert a dual role of action in living cells: First, it may represent a scaffold for the mTOR-S6K1 signaling axis, where it interacts with the inactive S6K1 in unstimulated conditions, while relieving it as phosphorylated S6K1 upon mTOR-mediated growth-promoting conditions. Second, eIF3 may solely act as a part of the translational preinitiation complex (PIC) and thus represent an essential component of the ribosomal protein synthesis machinery and cell size control. However, which of these two functions for eIF3 is presumably dominant over the other, remains currently unclear. To identify whether the observed hyperactive S6K1-S6 axis is the cause or consequence of eIF3-mediated reduction of cell size, we next evaluated the biological effects of a concurrent eIF3 and S6K1 inactivation. Therefore, we treated control and eIF3b/eIF3c-depleted IMR-90 cells with PF-4708671, a specific S6K1-inhibitor [35] for 24 hours. PF-4708671 treatment successfully inhibited the phosphorylation of ribosomal protein S6 in control and in eIF3b/c depleted cells (Figure 6A). To measure the effect of PF-4708671 on cell size, proliferation or apoptosis, we re-plated the non-treated, non-targeted and eIF3b/c-targeted IMR-90 for further 16 h in presence or absence of PF-4708671. As shown in Figure 6B, double knockdown of eIF3b and eIF3c significantly reduced the number of viable cells compared to non-targeted IMR-90 cells, yet no specific PF-4708671 effect was detectable. Similarly, PF-4708671 did not alter the difference in cell size caused by eIF3b/c-depletion (Figure 6C). When we analyzed the proportions of IMR-90 cells corresponding to distinct cell cycle and apoptotic stages, the eIF3b/c-depleted cells showed a profound increase in G1 and a concomitant decrease in S-phase, irrespective of the presence or absence of PF-4708671 (Figure 6D). To further substantiate these findings, we compared the cell size effects of PF-4708671 with rapamycin- and cycloheximide-treatments in each phase of the cell cycle. Treatment with rapamycin and cycloheximide led to a strong reduction of size in G0/G1-, S- and G2/M-phases, while PF-4708671 had no effect on size in any of the cell cycle phases (Figure 6E). This was not due to altered inhibitor activity since rapamycin and PF-4708671 effectively inhibited S6K1-activity (Figure 6F). Interestingly, S6K1-activity was increased in cycloheximide treated cells (Figure 6F), indicating that S6K1-activity *per se* does not correlate with size. To determine a possible direct effect of S6K1 on cell size, we next depleted S6K1 from IMR-90 cells and compared the size of eIF3b/c-depleted with S6K1-depleted IMR-90 cells. As shown in Figure 6G, eIF3b/c-depleted IMR-90 cells showed significant reduction of cell size in G0/G1-, S- and G2/M-phases, while S6K1-depleted cells did not. The corresponding knockdown efficiencies have been confirmed in Figure 6H. These findings suggested that specific S6K1-inhibition cannot modulate cell size of wild-type nor eIF3-depleted cells and that eIF3 regulates cell size independently of S6K1-activity.

**Figure 7:**
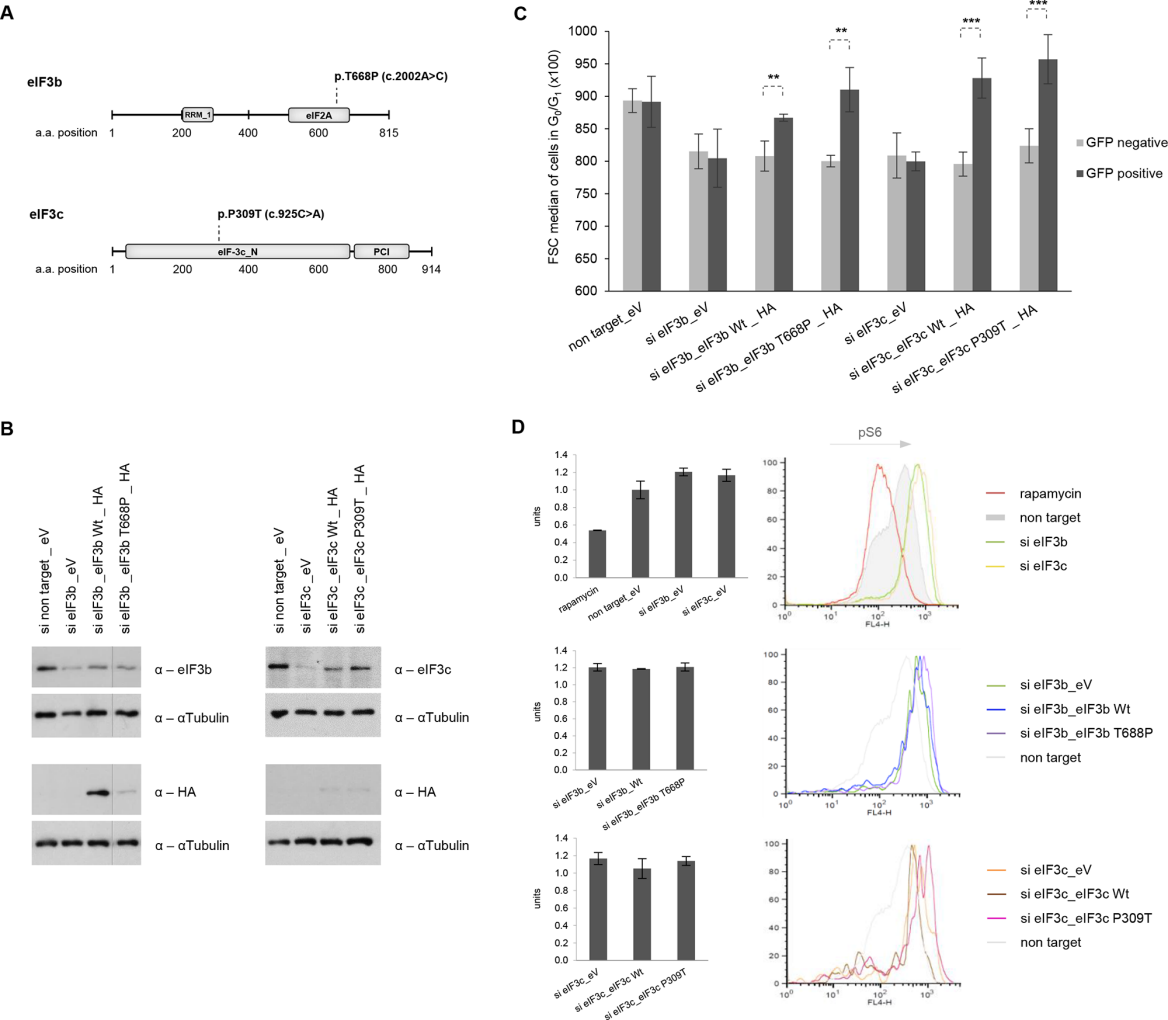
Re-expression of wild-type and mutant forms of eIF3b and eIF3c restores knockdown-induced cell size defects, independently of S6K1-activity **A.** Schematic representation of eIF3b and eIF3c protein secondary structure including annotated Pfam-A protein domains. The substitution missense mutation of eIF3b (p.T668P) is located in the eIF2A region and of eIF3c (p.P309T) in the N terminal region. RRM_1, RNA recognition motif; eIF2A, eukaryotic translation initiation factor eIF2A; eIF-3c_N, eukaryotic initiation factor 3 subunit 8 N terminus; PCI, PCI domain (Cosmic database). **B.** IMR-90 cells depleted for eIF3b and eIF3c were transfected with empty vector (eV), wild-type (Wt) eIF3b, Wt eIF3c or the corresponding mutants. To avoid interference in detection of eIF3b, eIF3c and HA-tagged proteins due to similar protein size, the same lysates were detected on separate membranes. **C.** A control vector carrying GFP-spectrin was co-transfected in all settings used. Cell size was measured in GFP-negative (untransfected fraction) and GFP-positive cells (transfected fraction). Representative bar diagrams out of two independent experiments are shown. Error bars correspond to means ± SD. **D.** Phosphorylation levels of S6 (S240/244) protein were determined by phospho-specific flow cytometry in GFP-positive cells using the same co-transfection setting as in (C) Representative bar diagrams and histogram overlays out of two independent experiments are shown.

The eIF3 complex has previously been associated with cancer initiation, since overexpression of particular eIF3-subunits has led to *in vitro* transformation of the immortalized murine cell line NIH-3T3 [5]. Our data suggested that eIF3b/c may play a pivotal role in the correct assembly and function of the eIF3-PIC complex, thereby influencing the cell size. This is underlined by the fact that translational control is the major regulator of cell size and that the failure to downregulate protein synthesis and presumably also cell size may lead to a malignant phenotype [5]. To provide additional support for this model, we sought to determine the relevance of lung carcinoma-associated single base pair mutations identified in eIF3b and eIF3c genes. To this end, we used the Catalogue of somatic mutations in cancer (COSMIC) library to derive two - yet uncharacterized - single base pair somatic mutations in eIF3b and eIF3c that have been confirmed in lung carcinoma. eIF3bT668P and eIF3cP309T were predicted to have an impact on the structure and function of eIF3b and eIF3c at the score >0.99 (Figure 7A). To determine whether both somatic mutations exert a gain-of-function or a loss-of-function mutation, we used wild-type and mutant eIF3b and eIF3c cDNAs cloned into the pcDNA3.1 mammalian expression vector. Successful overexpression of wild-type and mutant protein was determined using western blotting in IMR-90 and HEK293 cells (Figure S4A and Figure S4B). Next, we performed re-expression of wild-type and mutant proteins in the respective eIF3b and eIF3c-depleted background in order to clarify whether eIF3b and eIF3c constructs are capable of recovering the protein levels in knockdown cells. As shown in Figure 7B, we were able to re-express and partially revert the levels of reduced proteins in the respective knockdown background, using all wild-type and mutant eIF3b and eIF3c constructs. To test our model that the essential function of the eIF3 complex is to maintain cell size, we co-transfected wild-type and mutant eIF3b/c plasmids with GFP-spectin expression vectors into eIF3-depleted cells and analyzed the cell size of GFP-positive and GFP-negative cells by flow cytometry. As depicted in Figure 7C, the GFP-negative cell population has kept the reduced size phenotype of the eIF3b/c knockdown. Interestingly, the GFP-positive cells showed an increased cell size only in cases where wild-type or mutant eIF3b or eIF3c constructs had been used. However, we did not observe any difference in cell size between the cells carrying eIF3b wild-type and eIF3bT668P or eIF3c wild-type and eIF3cP309T constructs, respectively. We further asked whether re-expression of wild-type or mutant forms of eIF3b and eIF3c will influence the hyperactive S6K1-S6 signaling. We reasoned that if eIF3 regulated S6K1-activity, its re-introduction may be sufficient to reduce it; however, it is also possible that eIF3 cannot bind phosphorylated S6K1 [22]. Thus, we next measured the p-S6 levels in IMR-90 cells co-transfected with GFP-spectrin and eIF3b/c constructs using Facs analysis. Interestingly, high p-S6 levels (S240/244) could not be reduced by re-expression of any of the eIF3 constructs in knockdown background (Figure 7D, middle and lower panel). However, addition of 100 nM rapamycin for 1 h efficiently reduced the level of p-S6 compared to non-target siRNA and compared to knockdowns' p-S6 levels (Figure 7D, upper panel). Collectively, these findings show that eIF3 complex directly controls cell size and that S6K1-activity and cell size regulation via eIF3 are separable. Additionally, we show that cancer-associated eIF3bT668P and eIF3cP309T mutants positively regulate cell size and hence may not represent loss-of-function mutations.

## DISCUSSION

Cell size control is a fundamental cellular process requiring stringent external cues and high complexity of regulatory pathways. mTOR has been shown to regulate cell size in multiple cell systems and eIF3-complex and S6K1 have been suggested as key signaling molecules downstream of mTOR. Until now, it was unclear whether eIF3 complex and S6K1 have a joint role in cell size. We clearly show that depletion of either b or c subunit of the eIF3-complex leads to a decrease in cell size in normal, as well as, in transformed cells. Thus, we define a novel role for eIF3-complex in maintaining cell size, apart from regulating translational initiation. Unexpectedly, however, S6K1 does not participate in cell size control. To the best of our knowledge, these results implicate for the first time that cell size control may be regulated independently of S6K1-activity.

Recent evidence has suggested that eIF3b forms a complex with S6K1 only under conditions where S6K1 is not activated by phosphorylation at T389, whereas the phosphorylated S6K1 dissociates from eIF3b complex as soon as mTOR signaling is active [22]. In light of our data showing an increase in S6K1-activity upon eIF3-depletion, speculations can be made how this hyperactivity may have been achieved. Our results indicate that mTOR may be responsible for activation of S6K1 in absence of eIF3, since treatment with rapamycin was able to entirely abrogate S6K1 activity in eIF3-depleted cells. Importantly, alteration of the signaling downstream of hyperactive S6K1 did not have an effect on cell size. Specific inhibition of S6K1 signaling in wild-type or eIF3-depleted cells could not influence the cell size any further, indicating that neither increased nor reduced S6K1-activity has an effect cell growth. So far, studies on S6K1 in cell size regulation have yielded some contradictory results. Disruption of S6K1 in drosophila caused a reduction of cell size [16], however mice lacking S6K1 or S6K1 and S6K2 were viable, showed a body mass reduction (15–20%) despite remainder protein synthesis [17, 18]. RNA-mediated S6K1-depletion displayed little size reduction [21] and overexpression of S6K1 increased cell size [2]. Importantly, rapamycin resistant S6K1 mutants could only partially revert the size reduction induced by rapamycin treatment [2] and inhibition of S6K1-activity by PF-4708671 could not alter cell proliferation in a previous report [36]. Thus, how much of the S6K1-activity actually controls cell size might be cell type-specific. This is underlined by our finding that eIF3b shows distinct localization patterns in different cell lines and since eIF3 is the binding partner of S6K1, this imbalance may influence the extent to which S6K1 regulates cell size. Importantly, eIF3 regulated cell size irrespectively of the cell type, the species origin, the transformation status or the intercellular localization.

Indeed, S6K1 hyperactivity was accompanied by intracellular protein misplacement. mTOR-controlled nuclear localization of the p70 isoform of S6K1 has previously been reported [33]. Likewise, we have found a strong accumulation of nuclear p70 S6K1 in the current study. However, the increase in nuclear p70 S6K1 was dependent on the presence of eIF3-complex. A possible explanation for this finding is that eIF3 represents a retention factor of inactive p70 S6K1 expressed only in the cytoplasm, thus preventing it from shuttling to the nucleus. We were only partially able to substantiate this hypothesis, because eIF3 localized to both, cytoplasmic and nuclear fractions of IMR-90 and HEK293. It remains to be clarified by further studies whether or not eIF3 complex may shuttle dephosphorylated S6K1 back to the cytoplasm as a part of a recycling process for mTOR-mediated growth factor signaling.

The concomitant hyperactivity of S6K1 signaling and the reduced cell size reported in this study were clearly unexpected. However, similar “stimulation of S6K1-activity” has been reported already decades ago in chicken embryo fibroblasts, rat livers and Xenopus eggs following cycloheximide treatment [37–40]. Cycloheximide blocks the peptidyl-transferase reaction on ribosomes and is a potent inhibitor of translation, proliferation and presumably cell size [37, 40]. Interestingly, the increase in S6K1-activity reported in these studies has been explained as a proof for the existence of two independent growth-regulated signaling pathways that regulate protein synthesis [39]. Two possible mechanisms have been proposed to explain these observations: (i) lack of protein synthesis results in a lack of a negative regulator or stabilization of a positive regulator of S6K1-activity, or (ii) activation of a feed-back mechanism in an attempt to re-stimulate translation. Another explanation is that cycloheximide-induced increase in intracellular amino acids is responsible for the increase in mTOR-activity [41, 42]. In light of our data, it is tempting to speculate whether a lack of a downstream labile negative regulator - the eIF3-complex – may also cause a persistence of upstream signals from mTOR. Continued analysis of these signaling pathways will provide valuable information on how mTOR and eIF3 eventually regulate cell size.

Finally, we have been able to show that re-introduction of eIF3-complex – although unable to repress S6K1-activity – could efficiently rescue the size defects initially induced by its knockdown. Two lung cancer-associated mutations in eIF3b and eIF3c that have been tested in course of this study also had the capacity to reverse the reduced size phenotype, indicating that both mutations do not represent loss-of-function mutants of the eIF3-complex. Thus, it is highly likely that these mutations associate with a cancer phenotype in which the cancer cell size is normal or increased compared to the cancer cells without specific mutations. For lung carcinoma, this might correspond to non-small cell lung carcinoma (NSCLC) that are more differentiated and less prone to disseminate than the small cell lung carcinoma (SCLC) [43]. As both lung cancer-associated mutations described in this study were able to increase cell size of small (eIF3-depleted) cells, it may be speculated whether they associate with lung cancer types comprising of large cells – in this case NSCLC, rather than SCLC. Conversely, the second prediction from this data is that potential loss-of-function mutations in eIF3b and/or eIF3c may be associated with SCLC, rather than NSCLC. It remains to be determined by future studies whether these mutations are associated with advanced grade, stage or poor prognosis in lung cancer patients. Likewise, it remains to be clarified whether reduction of cell size through inhibition of eIF3-complex might represent a novel strategy against certain types of lung cancers.

In summary, our findings highlight the importance of eIF3-complex for cell size maintenance. In addition, we provide compelling evidence that size control may be independent of S6K1 activity. Thus, we propose that interference with eIF3-complex rather than S6K1-signaling will help determine new regulatory circuits that affect cell size in the future.

## METHODS

### Cells and cell culture

Primary human IMR-90 fibroblasts (#CCL-186) and human embryonic kidney HEK293 cells (#CRL-1573) were obtained from the American Type Culture Collection. MEFs were kindly provided by M. Pende. All cells were cultured in Dulbecco's modified Eagle's medium (DMEM) at 4, 5 g/l glucose, supplemented with 10% FBS and antibiotics (30 mg/l penicillin, 50 mg/l streptomycin sulphate) at 37°C and 5% CO2. For G0/G1 cell synchronization experiments IMR-90 fibroblasts were deprived of serum in growth medium containing 0.2% serum for 48 hours. Cells were stimulated to re-enter the cell cycle by the addition of 10% serum for another 36 hours. For p70 S6K1 localization experiments, cells were grown in DMEM supplemented with 10% serum for 60 hours. Cells were washed with 1xPBS following the addition of fresh DMEM supplemented with 10% serum to one set of cells and 0% serum to a second set of cells for 12 hours. Cells were then harvested and proteins of specific cellular fractions analyzed via immunoblotting.

### siRNA transfections

The following ON-TARGETplus SMARTpool siRNAs from Dharmacon were used: human eIF3b (#L-019196–00), human eIF3c (#L-009036–00), human mTOR (#L-003008–00), human S6K1 (#L-003616–00) or for non-sequence specific effects non-targeting siRNA control pool (#D-001810–10). siRNA transfection experiments were carried out using Lipofectamine RNAiMax transfection reagent (Life Technologies) according to the manufacturer's instructions. In experiments where simultaneous knockdown of two genes was performed, the overall amount of siRNA for each reaction was kept constant by the addition of non-targeting siRNAs. Cells were seeded in 6-well plates at a confluence of around 20–30% and transfected 12–16 hours later at a final concentration of 50 nM. 72 hours post transfection cells were harvested and analyzed if not otherwise stated. For re-plating experiments like cell cycle experiments, confluent cells were re-seeded 72 hours post transfection and analyzed 16–20 hours later.

### Identification of eIF3b and eIF3c mutations

Using COSMIC, specific somatic mutations of the eIF3b and eIF3c genes derived from tumor samples were analyzed for the type of mutation and its localization [44]. Furthermore, the PolyPhen-2 (Polymorphism Phenotyping v2) online tool software was used to obtain a possible prediction of the impact on the function and structure of the protein caused by the defined somatic mutations. Non-synonymous mutations, localized at protein interaction sites and with a high PolyPhen-2 prediction score were selected for our analysis [45]. We conceived plasmids including the wild-type cDNA sequence of eIF3b or eIF3c and furthermore vectors with the defined single base-pair substitutions as identified in selected mutations from COSMIC.

### Plasmid constructs and transfections

eIF3b and eIF3c wild-type and mutant plasmids were generated and purchased by Invitrogen: HA-eIF3b wild-type, mutant HA-eIF3bΔT668P, HA-eIF3c wild-type and mutant HA-eIF3cΔP309T, all cloned into pcDNA3.1 vector. DNA transfection experiments were carried out using Lipofectamine 2000 transfection reagent (Life Technologies) according to the manufacturer's instructions. Cells were seeded in 6-well plates at a confluence of around 70–80% and transfected 12–16 hours later with 1 μg plasmid DNA. Cells were harvested 24 hours post transfection and analyzed via immunoblotting.

For combined approaches of siRNA mediated knockdown and plasmid re-expression cells were transfected with siRNA for 2 or 48 hours following DNA transfection for another 72 or 48 hours, respectively. Of note, both protocols did not yield any difference in the expression of the desired proteins. For plasmid re-expression, cells were additionally transfected with GFP-spectrin expression vector as a reporter at a final ratio of 1:5 [46]. Cells were harvested and analyzed via immunoblotting and flow cytometry.

### Inhibitor treatments

Cycloheximide (Calbiochem, #239764) was added to the cells for 3, 5 hours at a final concentration of 50 μM to inhibit protein synthesis. mTOR specific inhibitor rapamycin (Calbiochem, #553211) was used at a final concentration of 100 nM. Knockdown cells were treated with rapamycin 48 hours post transfection for 24 hours. Control cells for phospho-specific flow cytometry were treated with rapamycin for 1 hour. p70 ribosomal S6 kinase (S6K1) inhibitor PF-4708671 was used at a final concentration of 10 μM. 48 hours post siRNA transfection PF-4708671 was added to the cells for 24 hours. As cells reached confluence 72 hours post siRNA transfection, passaging of cells was performed. PF-4708671 was added to the new cell culture medium for further 16 hours. For cell size measurements in each cell cycle phase, IMR-90 cells were treated for 24 hours with the following concentrations: 100 nM rapamycin, 10 μM PF-4708671 and 20 μg/ml cycloheximide. DMSO was used for treatment of control cells in place of the inhibitors.

### Cell count

Cells were harvested by trypsinization and single cell suspensions subjected to the Casy TTC cell counter (Roche) evaluating cell number (cell count) and overall cell size (volume).

### Flow cytometry

Single cell suspensions were either fixed with ice-cold 85% Ethanol, 4%PFA/100%Methanol or Foxp3 Fixation/Permeabilization kit (eBioscience, #00–5521-00) as appropriate or previously described [47]. DNA was stained with propidium iodide staining solution (0.25 mg/ml propidium iodide, 0.05 mg/ml RNase, 0.1% Triton X-100 in citrate buffer, pH 7.8). For intracellular staining primary p-S6 S240/244 specific antibody (Cell Signaling, #5364) was used at a final concentration of 1:200. Following second step antibodies were used at a final concentration of 1:500: APC-labeled goat anti-rabbit IgG antibody (Invitrogen, #A10931) or Alexa Fluor 647 conjugated goat anti-rabbit IgG antibody (Cell Signaling, #4414). Cells were analyzed on a FACS Calibur or FacsCanto II flow cytometer (Beckton Dickinson, New Jersey, USA) and corresponding data were analyzed using FlowJo or CellQuest Pro software. FSC values, representing means or medians were obtained using CellQuest and FlowJo histogram statistical tools. GFP-spectrin positive cells, representing plasmid re-expressing cells, were selectively gated for further analysis.

### Immunoblot analysis

Cells were harvested and whole cell, cytoplasmic and nuclear fractions were prepared as described previously [33]. Equal amounts of protein lysates were resolved on SDS-PAGE, transferred to nitrocellulose membranes and detected via immunoblotting using antibodies specific for the following proteins: eIF3b (Bethyl, #A301–761A), eIF3c (Bethyl, #A300–377A), 4EBP1 (Cell Signaling, #9644), mTOR (Cell Signaling, #2972), S6 (Cell Signaling, #2317), S6 S240/244 (Cell Signaling, #2215), p70 S6K1 (Cell Signaling, #9202), p70 S6K1 T389 (Cell Signaling, #9205), CAD (Cell Signaling, #12662), Akt S473 (Cell Signaling, #4060), PDCD4 (Cell Signaling, #9535), Fibrillarin (Cell Signaling, #2639), HA (Cell Signaling, #3724), Cyclin A (Santa Cruz, #sc-751), Cyclin D1 (Santa Cruz, #sc-718), p27^(Kip1)^ (BD Biosciences, #K25020), GAPDH (Trevigen, #2275-pc-100) and αTubulin (Calbiochem, #CP06). The following secondary antibodies were used: anti-rabbit IgG, HRP-linked heavy and light chain antibody from goat (Cell Signaling, #7074) and anti-mouse IgG, HRP-linked heavy and light chain antibody from horse (Cell Signaling, #7076). Signals were detected by chemiluminescence (Pierce, #32106).

### Nascent protein synthesis

72 hours post siRNA transfection, new protein synthesis was evaluated using Click-iT metabolic labeling reagent L-azidohomoalanine (AHA) and the Click-iT protein reaction buffer kit (Life Technologies) according to the manufacturer's instructions. Cells were washed twice with 1xPBS and cultured with Methionine/Cystein free medium for 1 hour. AHA was added to the cells at a final concentration of 50 μM for 3.5 hours and detected by tetramethylrhodamine (TAMRA) (Life Technologies) via the Click-iT reaction. Protein lysates were run on SDS-PAGE and gels scanned for fluorescence (TAMRA; Ex545/Em580) using a Typhoon scanner. Gels were transferred to nitrocellulose membranes and by immunoblotting, total protein loading was evaluated by using αtubulin primary antibody.

### Statistical analysis

At least two independent experiments including triplicates were performed for each experiment. Results are shown as means ± SD where applicable. For statistical analysis, the significance of the observed differences was determined by using Student's *t-test* (two-tailed, unpaired). *P*-values ≤ 0.05 are defined as statistical significant.

## SUPPLEMENTARY INFORMATION


