# Supplementary material for: eIF3 controls cell size independently of S6K1-activity
Source: Oncotarget. 2015 Jun 29;6(27):24361–75. doi: 10.18632/oncotarget.4458 (PMC4695191; doi:10.18632/oncotarget.4458)
Supplement: Supplementary file 1 [file oncotarget-06-24361-s001.pdf]

SUPPLEMENTARY INFORMATION

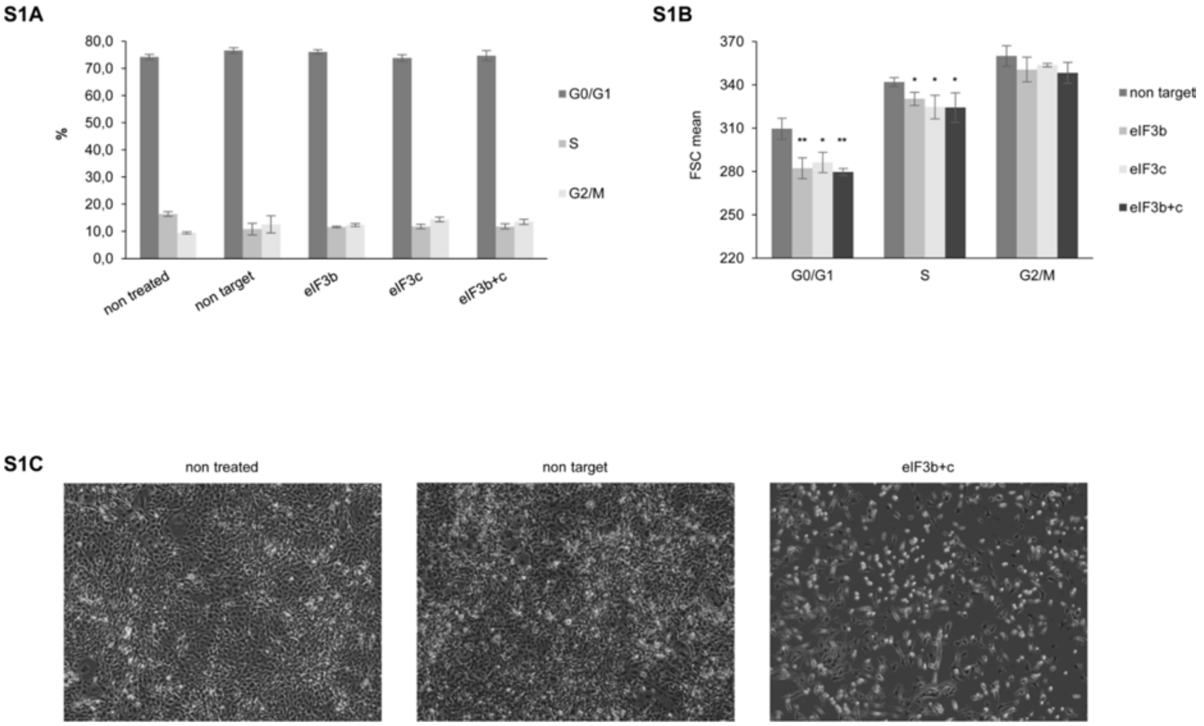

Supplementary Figure S1: eIF3b+c knockdown effects on cell cycle distribution and cell size in IMR-90 cells; effects on cell density in MEFs. IMR-90 cells were transfected with specific siRNAs or left untreated as indicated for 72 hours and analyzed for **A.** cell cycle distribution and **B.** cell size specifically for each phase of the cell cycle by flow cytometry using the parameter FSC. (**A** and **B**) One representative experiment out of three independent experiments performed in triplicates is shown. Error bars correspond to means ± SD. **C.** MEFs were transfected with non-targeting control siRNA, eIF3b and eIF3c specific siRNAs. Representative pictures of cultured MEFs at 72 hours post transfection are shown (magnification 4x).

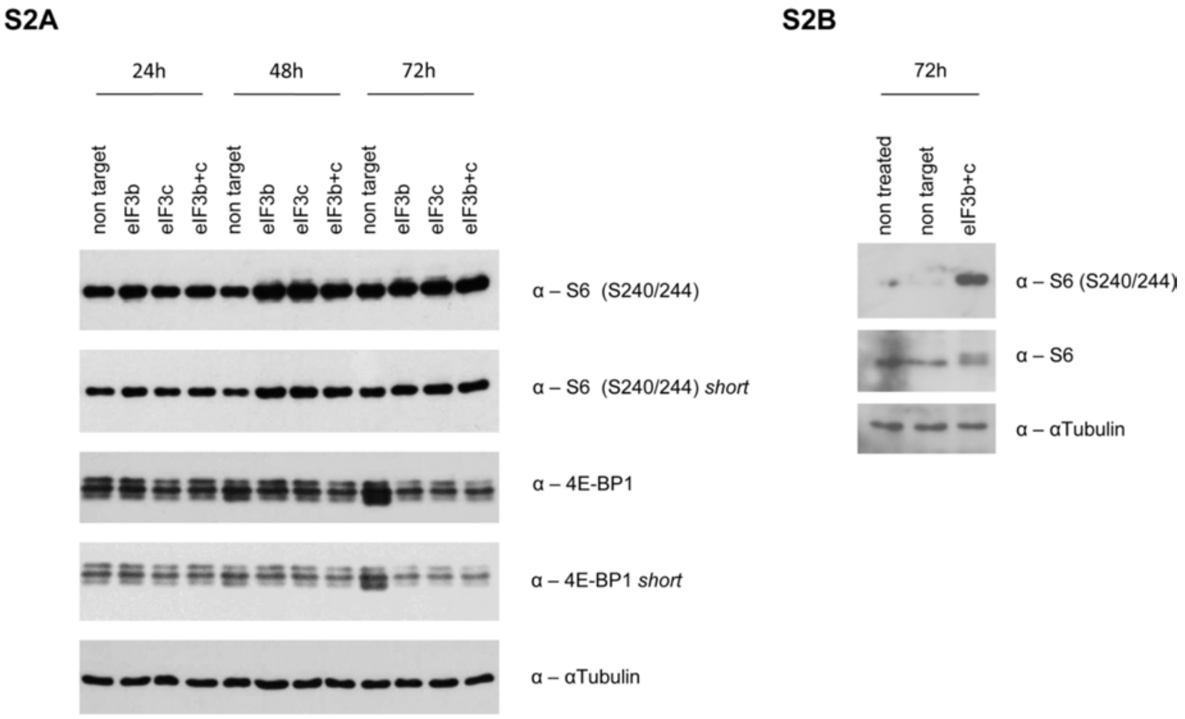

Supplementary Figure S2: Increased S6K activity upon eIF3b/c knockdown is already present 48 hours post transfection. A. Human IMR-90 fibroblasts were transfected with non-targeting control siRNA, eIF3b and/or eIF3c specific siRNAs. IMR-90 cells were harvested 24, 48 and 72 hours post transfection. Protein lysates were analyzed for mTORC1 downstream targets. αTubulin was used as a loading control. **B.** Phosphorylation levels of S6 (S240/244) in MEFs were verified by immunoblotting. αTubulin was used as a loading control.

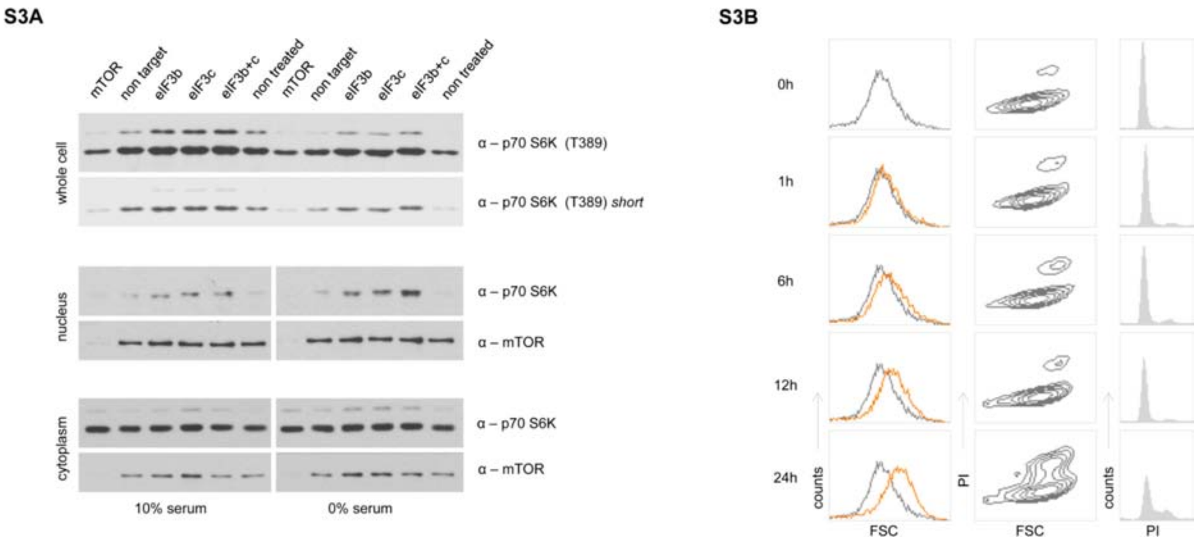

Supplementary Figure S3: Knockdown of eIF3b, eIF3c or eIF3b+c induces nuclear p70 S6K. A. IMR-90 cells were transfected with siRNAs specific for mTOR, eIF3b and eIF3c in single and double knockdowns, non-targeting control or left untreated for 60 hours. Cells were stimulated with 10% serum or serum-deprived for additional 12 hours. Expression levels of p70 S6K, phospho-p70 S6K (T389) and mTOR were analyzed by immunoblotting after fractionation as indicated. **B.** Control flow cytometry profiles of IMR-90 cells after re-stimulation. IMR-90 cells were cell cycle-synchronized in G0/G1 via serum deprivation and then re-stimulated by adding 10% serum. Using flow cytometry, the re-entering into cell cycle upon serum re-stimulation was determined at 0, 1, 6, 12 and 24 hours. Cell size (FSC) and cell cycle distribution (PI) are shown.

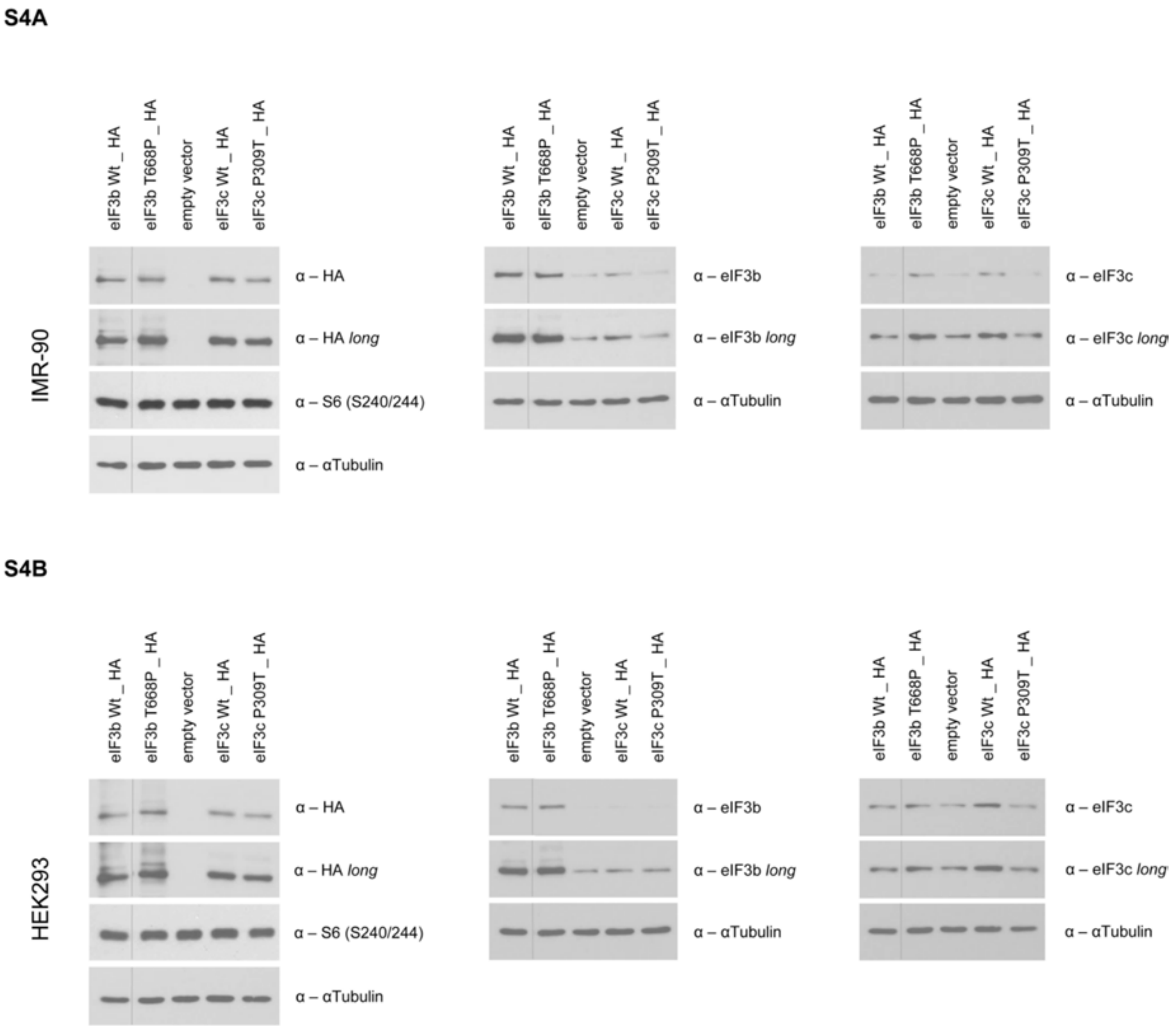

Supplementary Figure S4: Overexpression of wild-type and mutant eIF3b and eIF3c plasmids in IMR-90 and HEK293 cells. Cells were transfected with empty vector (pcDNA3.1), wild-type (Wt) eIF3b, Wt eIF3c or the corresponding mutants for 24 hours. **A.** Cell lysates of IMR-90 cells were immunoblotted with indicated antibodies. To avoid any interference in the detection of HA, eIF3b and eIF3c due to similar protein size, same lysates were detected on separate membranes. **B.** Overexpression of eIF3b and eIF3c wild-type and mutant plasmids as in panel (A) was also verified in HEK293 cells.
